# The Origin, Nature and Treatment of Fever, Etc.

**Published:** 1873

**Authors:** Ch. Rauschenberg

**Affiliations:** Atlanta, Ga.


ATLANTA

### MEDICAL AND SURGICAL JOURNAL.

VOL. XI.—SEPTEMBER, 1873.—NO. 6.

### ORIGINAL COMMUNICATIONS.

### THE ORIGIN, NATURE, AND TREATMENT OF FEVER IN THE LIGHT OF MODERN PHYSIOLOGY.

BY CH. RAUSCHENBERG, M.D., ATLANTA, GA.

(*Continued from August Number.*)

Thus, fever originates in more or less intense blood-changes, and its degree, character, and duration depend primarily upon the quantity, quality, and duration of the fever cause.

A morbidly increased, general oxygenation and disintegration of the blood and tissues—a general morbid increase of the metamorphosis within the human system—primarily the result of peripheric vaso-motor irritation by the abnormal blood, and *characterized by an increased temperature of the body*, constitute the essence of the febrile process.

Irritation of any other part of the nervous system can not directly produce fever. The manifold and often grave cerebro-spinal disturbances of hysteria, for instance, may, through the brain and spinal marrow, produce functional abnormities of the vaso-motor system of nerves. A hysterical woman may have a frequent pulse and a flushed countenance, and at the same time a cool skin; hence no fever, because the fever cause within the blood itself is absent. A so-called irritative fever is therefore an untenable pathological conception. Irritation of any portion of the cerebro-spinal system can never produce fever unless it affects the vaso-motor or trophic nerves of an organ of the system by reflex action, in such a manner as to produce local functional derangement or local inflammation, and thereby again chemical or histological alterations in the blood, and thus fever secondarily, in the manner above discussed.

The metamorphosis in fever, however, is principally of a retrograde character. The functions of digestion and assimilation, in consequence of sympathetic participation of the organs by which they are carried on, become deranged at an early stage of the febrile process, and hence, as new material is but scantily furnished, the chemical affinities governing this metamorphosis are principally exercised in a retrograde direction, and the organic material of the system is consumed by an increased production of carbonic acid and water on the one hand, and urea, uric acid, kreatin, etc., on the other.

The interpretation of the febrile process of Booerhave, Sydenham, Stahl, and others, mentioned in the commencement of this paper, as a salutary and—so to say—physiological effort of nature to eliminate the very substances from the system which have given rise to it, is therefore entitled to a just degree of consideration.

Where these noxious substances in the blood, which have instigated the fever, are of such a character that they can be converted into excretory matter, and as such or with it removed from the system without causing too high a degree of and too long-continued combustion of the organic material of the system, the febrile process will terminate favorably; where this conversion and elimination excites a higher degree of temperature of the blood, or larger amount of combustion of material than the system can bear, death takes place before its termination. A correct conception of the degree of the temperature developed in each case of fever, and the degree of the consumptive effect in each case upon the system generally, are, therefore, of the greatest therapeutical importance to the practicing physician.

The great clinical importance of abnormal grades of temperature in diseases, and the high grades of it in fevers generally, will be more fully comprehended if the following physiological and pathological facts are taken into consideration:

*The temperature of the human body in health is the same under*

*all latitudes and in all climates, and this permanency of temperature is an absolute necessity for the normal physiological action of all its elements and organs, and therefore for the continuation of health and life.*

The average temperature of men taken at the axilla is 98.6 F., or 37° C. The human body must, therefore, necessarily have the faculty to maintain its specific degree of temperature permanently, independent of the changes in the temperature of the surrounding medium, and the different quantities of caloric produced within the blood and tissues at different times, or, in other words, the production and loss of caloric of the human system must regulate each other mutually; a high grade of production must always correspond with a high grade of loss, and so vice versa. Any material rise or fall of the bodily temperature above or below the normal standard can not exist any length of time without serious danger to the system: 35° C., (95° F.) and 42.5° C. (108.5 F.) are the extreme deviations of temperature observed in serious cases of disease; a decrease of 1.5° F., and an increase of 9.9° F., are deviations from the normal standard, which, according to undeniable observations, are almost infallibly connected with the death of the patient. If an increase of about 10° F. is a certain indication of early dissolution, it is quite reasonable to suppose that the intermediate grades of increase are connected with a corresponding degree of danger. Experience as well as observation support the fact that human blood, *as soon as its temperature rises beyond the normal degree, begins to undergo histological changes, which make it unfit for healthy nutrition, in direct proportion to the increase of its temperature.*

Schultze* has microscopically demonstrated these changes in their full development on blood heated on an objective table to 50°C., a temperature which the human blood in life never reaches; but his experiments establish beyond a doubt the disintegrating effect of heat as a physical agent on blood, and that this disintegration commences as soon as its temperature begins to ascend beyond the normal degree, is at least a very natural conclusion, and in full accordance with the general action of all laws of nature.

* See Atlanta Medical and Surgical Journal, vol. x, page 296.

It is also a fact, well established by clinical observation, that the reparative process required for the healing of wounds or ulcers is considerably or entirely suspended by the occurrence of febrile diseases.

Liebermeister* has seen chancroids become phagedenic in individuals who were taken with typhoid fever, and every physician has seen wounds cease to granulate and assume an unhealthy appearance under similar circumstances.

The anatomical condition of the different organs of patients who died under the influence of high grades of fever, revealed by the microscope, furnishes, finally, the most conclusive evidence of the decomposing and disintegrating effect of the febrile combustion on the tissues, and the plainest explanation of the deleterious influences of high febrile temperatures on the human system.

Numerous post-mortem examinations of fever patients have proven beyond a doubt, that those of them who died under the influence of a high grade of temperature, *and they alone*, exhibited parenchymatous degeneration of the most important organs, while those cases where such a high elevation of temperature did not occur, were free from them.

Thus the cellular elements of the liver are always found more or less plainly degenerated, sometimes entirely decomposed into loose detritus. Similar changes are observed in the kidneys. The heart is unusually loose, flabby, of a paler, grayish yellow or brownish color, and in the primitive muscular fibres, granular opacity, indistinctness of the transverse stripes, and accumulation of fat granules are often plainly perceptible. The same changes and also waxy degeneration are often observed in the voluntary muscles. Similar degenerations are noticed in smaller blood vessels, in the substance of the brain, and many other parts of the organism.

These changes are spoken of by Prof. R. Virchow, in the 16th chapter of his Cellular Pathology, as the highest grade of nutritive irritation (parenchymatous inflammation in his sense of the term) and as processes in which by the increased activity of the cellular elements of different organs an abnormal quantity of nutritive material is attracted but not converted into normal

*Uber die Behandlung des Fiebers. Volkman's Klin Vortraege, No. 31, p. 214.

cell-substance, and which therefore terminate either in softening (necrobiosis) or in fatty degeneration.

He speaks of them as very frequent and very serious pathological conditions, occurring principally in infectious diseases: the acute exanthemata (measles, scarlatina, variola,) typhus, puerperal and traumatic fevers, phlegmonous and erysipelatous processes, and other blood intoxications. Prof. C. Liebermeister of Basel, one of the most prominent investigators of temperature conditions in health and disease, and an acknowledged authority on these subjects, ascribes the frequent occurrence of these local parenchymatous degenerations in infectious disease, not to their infectious origin, but to the high grades of febrile temperature generally prevailing during their entire course, because these morbid anatomical conditions are alike observed in all cases of disease which terminate fatally, under very high grades of febrile temperature, whether they are of an infectious character or not.*

*This deleterious influence of high febrile temperatures on the tissues constitutes one of the great dangers connected with acute febrile diseases per se*, and manifests itself principally, as it disturbs the heart or the cerebro-spinal centers prominently, by two trains of symptoms which terminate respectively in paralysis of the heart or the brain.

An increasing frequency and weakness of the pulse, corresponding deficiencies of the capillary circulation, hypostatic troubles, cold skin and extremities, with increased internal temperatures—symptoms frequently called congestive—mark the former; delirium and versatility, followed by stupor, coma, insensibility, the latter condition of things, wherever a high febrile temperature continues for a longer time, the disease itself may be scarlatina or erysipelas of the face, pneumonia or acute rheumatism, or any other acute febrile condition. When practitioners speak of acute febrile diseases assuming an adynamic or typhoid type, they are generally dealing with the deleterious consequences of high febrile temperatures on the tissues of the heart or nervous centres, and the grave functional disturbances which follow them. The most malignant forms of these diseases and their fatal terminations or their serious local se-

*C. Liebermeister. Uber die Behandlung des Fiebers. Volkmann's Smulung Klinischer Vortraege, No. 31, 1871.

quelæ—for instance, the occasional malignancy of scarlatina, erysipelas, etc.—are caused by these structural degenerations of important organs. An acquaintance with these occurrences and their dependence upon high febrile temperatures is of great diagnostic and therapeutical importance. It demonstrates to the practitioner the great value of thermometrical observations in all febrile and inflammatory diseases, and the great necessity of controlling the temperature of the body in fevers in order to prevent the occurrence of these conditions, and enables him, finally, to explain severe circulatory and cerebral disturbances frequently without resorting to the supposition of meningeal inflammation or uræmic intoxication, etc.

*Another not less serious danger, directly resulting from the increased combustion and matamorphosis constituting the febrile process per se, consists in the morbid consumption of the organic material of the body and its subsequent depressing influences on the functions of the brain and nervous system.* Every fever is a consumptive process, and deserves more or less the name of a hectic fever.

While in acute febrile diseases the high grade of febrile temperature causes the parenchymatous degeneration of important organs, in low continued fevers the long duration of a less excessive febrile temperature sets up a waste of tissue material, which will destroy life in due time as certain as the former condition.

A correct appreciation of the therapeutical importance of these pathological facts, the unavoidable results of the very nature of the febrile process per se, independent of its specific character, its local origin or complications,has initiated the most essential advances of modern clinical medicine in the treatment of febrile diseases. It has taught the profession to recognize, by the use of the medical thermometer, the difference between a febrile condition, which is destitute of danger and will terminate in health, and one which, in consequence of the high or too long-continued febrile temperature, consumption and disintegration of organic material, excludes the possibility of the continuation of the various processes of life, and has established: *reduction and control of high febrile temperatures as the most prominent and most important general indication in the treatment of all febrile conditions.*

The necessity for a more exact measurement of febrile temperatures than the mere subjective sensation of the observer can furnish, has been felt for a considerable length of time, and De Haen, J. Currie, and other isolated investigators have already used the thermometer for that purpose. The clinical importance of a knowledge of the physiological laws by which the production and consumption of caloric in the human system is governed in health and in disease has also been apprehended, and numerous physiologists and pathologists—Sanctorious, of Padua, De Haen, of Vienna, Ch. Martin and Hunter, in England, Lavoisier and Laplace, in France, James Currie and others—have investigated and published their physiological observations on this subject; but the introduction of regular thermometrical measurements of the temperature in diseases as an important auxiliary measure in establishing a correct knowledge of each case, its prognosis and treatment belongs to the very latest period of time.

Andral, Rogers, Demarquay, and others, have, since 1840, paved the way by valuable observations in this direction, but Berensprung, Traube, and most prominently, Wunderlich, of Germany, are the real founders of clinical thermometry. The latter, more than any one else, has established the use of the medical thermometer as an indispensable necessity to arrive at a correct understanding of the prognosis, nature and treatment of febrile diseases, and has elevated thermometry to an important branch of pathology and therapeutics. The doubts existing in the minds of many physicians yet as to the practical value of thermometry will be dispelled if they will take the pains of studying Wunderlich's and Seguin's Medical Thermometry, published by Wood & Co., New York, 1871. Wunderlich, on page 25 of that book, calls thermometry: An objective physical method of investigation which gives, earlier than all other methods, a more accurate and delicate measure of the changes in the organism, which may be graphically expressed by traces, or numerically by figures, and says: "Thermometry is a part of our method of diagnosis or observation of disease, indispensable where temperature varies, useful in doubtful cases, and auxiliary in almost every case. *A medical attendant who undertakes to decide a case of fever or febrile disease without knowing the facts of thermometry is like a blind man trying to find his way in an unknown locality.*"

This expression may sound harsh and perhaps extreme, particularly to those who are yet practicing medicine without ever using a thermometer; but an observer like Wunderlich, who studied 25,000 cases thermometrically, and made careful records within their history of several millions of single thermometrical observations before he published his work, has a right to speak definitely on the subject.

It is beyond the reach of this paper, which considers only the general pathology and treatment of the febrile process, to enter into detailed statements of the many important data which this late field of observation has furnished in relation to differential diagnosis, prognosis and treatment of various forms of febrile diseases. It is, however, one of the objects of it to impress the fact upon the mind of the profession that febrile diseases cannot be treated rationally and in harmony with the principles of modern pathology without the aid of the medical thermometer and an acquaintance with the facts of thermometry, because only that instrument enables the practitioner to ascertain the degree of fever and the amount of danger connected with it. It is the measure by which the time *when*, and *the degree of energy and perseverance* can be determined with which, the now accepted antifebrile measures should be used for the prevention or amelioration of the above discussed dangers of fever, better than by all the other symptoms together, although no intelligent practitioner should, on that account, neglect a careful consideration of the condition of every part and function of the organism and the relationship and influence, which they mutually occupy to, and exercise upon, each other.

Thus the thermometer is as great an aid to the physician and furnishes him as unmistakable indications in the treatment of disease as the compass does to the mariner, and its introduction into the practice of medicine has been and will continue to be followed by *alike important* and beneficial results.

The most important general indication in the treatment of the febrile process has already been defined as: *reduction and control of the temperature according to the data furnished by thermometrical measurement.*

This is the only indication which can be obtained by a careful study of the general pathology of that morbid process, and therefore a discussion of the question by what modes of treatment, and by what remedies it can directly be fulfilled, will form the only therapeutical consideration of this paper.

The most direct anti-febrile or—as it is now more frequently designated—anti-pyretic mode of treatment consists in the application of cold water to the hot body of the fever patient by which a portion of its caloric is consumed and withdrawn.

Physicians have in all ages, governed by the desire of their patients for cool drinks, cool air, etc., been more or less inclined to use cooling remedies, frequently, however, more for the comfort of the patient than with a view of obtaining marked therapeutical results. The application of cold water or ice to the head was, until quite recently, the most energetic remedy ever used for the purpose of reducing heat, and very few physicians would have ventured to use cold applications to the thorax o abdomen in [fevers, much less systematic reduction of the febrile temperature by frequent immersions of the entire body into cold baths.

James Currie, an English physician, was the first who, near the end of the last century, used and introduced the systematic treatment of febrile diseases by cold water for the purpose of reducing the febrile temperature. His method found some successful imitators; but was soon forgotten.

Priesnitz and his followers, the founders of the hydropathic treatment par excellence, used cold water for almost any other purpose but that of simply reducing the temperature of the body, and very few physicians, during the first half of our century, had any confidence in its use in febrile conditions. It was reserved to Dr. Ernst Brandt, of Stettin, Germany, to rehabilitate this method by his work on the treatment of typhoid fever.

Since that time it has gained ground rapidly, and has been extensively used and carefully studied by numbers of the most prominent pathologists of Germany. Bartels and Juergensen, of Kiel, Liebermeister, of Basel, Wunderlich, of Leipsic, Gerhardt, of Jena, Drasche, of Vienna, Ziemsen and Immerman, of Erlangen, Pfeufer, of Munich, and many others, have introduced this method in their hospitals, and have treated thousands of cases of enteric fever (abdominal typhus), one of the most common diseases of Germany, in this manner.

They have all published the results of their investigations, and are all more or less enthusiastic advocates of the antipyretic treatment, as it has not only reduced the mortality of that disease greatly, but has a decided and marked influence in checking or ameliorating its various local symptoms. Both facts have been statistically demonstrated.

Liebermeister gives the following statements in relation to the mortality of the disease under different modes of treatment at the hospital at Basel.

1. Under expectant and symptomatic treatment:

| Year. | No. of Cases. | Death. | Percentage of Mortality. |
|---|---|---|---|
| 1843—1853 | 444 | 153 | 30.4 |
| 1854—1859 | 643 | 172 | 26.7 |
| 1860—1864 | 631 | 162 | 25.7 |

2. Under imperfect antipyretic treatment:

| | | | |
|---|---|---|---|
| 1865—1866, Sept. | 982 | 159 | 16.2 |

3. Under energetic and continued antipyretic treatment:

| | | | |
|---|---|---|---|
| 1866, Sept.—1867, (end) | 339 | 33 | 9.7 |
| 1867—1868 | 181 | 11 | 6.1 |
| 1868—1869 | 186 | 10 | 5.4 |
| 1869—1870 | 139 | 10 | 7.6 |
| | 845 | 64 | 7.6 |

Dr. Brandt's reports show the same favorable decrease of mortality from 30 per cent. to 10 per cent.; Stöhr's in Würzburg from 31.5 to 6.6 per cent.; Ziemsen's from 15.4 to 3.1 per cent.

Liebermeister's experiments have further established the fact 1. That abstractions of caloric from the body of fever patients by cold baths, cause first an increased combustion followed after the bath by a decrease of combustion and temperature much greater than the preceding increase, and therefore more than compensating its effects.

2. That this decrease of combustion and temperature caused by the cold bath depends upon the duration and temperature of the latter. The colder the water and the longer the immersion, the more decided the refrigerating effect; a bath of short duration but sufficiently cold, has a more decidedly cooling effect than a bath of a few degrees higher temperature much longer continued.

To Juergensen and Bartels in Kriel, belongs the merit of having demonstrated that in a very large majority of febrile patients intensely cold baths can be used as often as needed, or in other words, as often as the febrile temperature rises to 39 degrees C. (102 deg. F.) without any danger. From the introduction of this energetic method of treatment (1866,) dates the great diminution of the mortality of abdominal typhus set forth in the above statistical statements.

The leading practical points for the guidance of the practitioner in the use of this method of treatment are:

1. Cold water as a refrigerant should be used in all cases of disease where the grade of the bodily temperature—39 deg. C. (102 deg. F.) or above—threatens to initiate the above mentioned dangers of the febrile process. Abdominal typhus, measles, scarlatina, variola, pueperal fever, epidemic cerebro-spinal meningitis, bronchitis, severe forms of pneumonia, etc., frequently require this treatment.

2. The success of it depends upon the energy and perseverance with which it is adapted to the indications which each individual case presents. The course of the temperature, the condition of the pulse, the respiration, and particularly the strength of the patient, must be carefully consulted, in order to decide what amount of benefit can be expected from abstractions of caloric by the use of cold baths or cold applications. In anæmic and already much emaciated individuals and in diseases of a chronic character, for instance phthisis, its use is of doubtful propriety, as the increase of combustion during each application might, in some cases, do more harm than the subsequent decrease of it would do good.

3. Whenever its use is deemed proper, it should be resorted to *early and energetically* to obtain the most favorable results. Baths and other cold applications should be used—not promiscuously and in irregular intervals—*but as often as the febrile temperature measures in the axilla* 39 *deg. C.* (102 *deg. F.*) or above.

Full baths in water of 20 deg. C. (68 deg. F.) of about ten minutes duration, are used by Wunderlich, Liebermeister, Ziemsen, and others, as the most effective antipyretic application. The bathing tub remains in the sick chamber and the same water is used during the entire day for one patient. In hot weather it is cooled down by the addition of ice if needed. Weakly patients are kept in it only from 5 to 7 minutes at a time. After the bath the patient is at once put to bed, wrapped up in a dry cloth, without being previously tired by an effort of wiping his body, and covered lightly. If shivering, he takes a glass of good wine and his feet are kept moderately warm by suitable applications. In very serious cases the treatment is commenced with baths of a higher temperature—24 degrees C. (72.2 deg. F.) or with baths of 35 deg. C. (95 deg. F.) gradually cooled down by the addition of cold water, but continued each time as long again as colder baths.

Applications of wet cold sheets around the entire body, leaving only the feet free, are, next to baths, the most effective mode of reducing the febrile temperature. Three to four consecutive applications of from 10 to 20 minutes duration each, produce the same effect as one cold bath. This method recommends itself in private practice on account of its simplicity. Winternitz uses the sheets without any cover over the patient, and continues to sprinkle cold water on the sheets where a very high temperature requires it, and uses frictions on parts which remain unusually cool, a condition of things occasionally occurring.

In cases running their course with mild febrile temperatures and less intense nervous symptoms, or in children where the amount of the surface in proportion to the weight of the body is always more considerable than in grown persons, this method of abstracting caloric is very applicable and often quite sufficient.

Cold douches as well as ablutions frequently repeated can, particularly in cases of sopor, etc., where an exciting effect on the psychical and respiratory functions is desired, be made useful, but as refrigerants they are, according to repeated calorimetric measurements, of very little lasting benefit, and no substitutes for cold baths or cold sheets.

This method of treatment reduces the temperature of the body indirectly by the physical abstraction of caloric from its surface. Its energetic use is often sufficient to diminish the febrile temperature more or less permanently, and to obviate the dangers directly resulting from its continuation. Where this is not the case, or where this treatment is not well endured by the patient, or where the already existing high grade of bodily consumption and weakness as in phthisis forbids its energetic use, other additional remedies are resorted to, which by their internal influence on the blood or nervous system exercise a direct controlling effect on the production of caloric.

Quinine and veratrum viride are, next to cold water, the principal agents for the reduction of febrile temperatures. The former remedy is now extensively used in connection with the cold water for the accomplishment of this purpose. *One large dose from* 20 *to* 40 *grains in adults is given at once, or within the course of one hour, and repeated as often as the reduced temperature rises again, which is seldom the case before* 48 *hours from the time of the administration of the remedy.* If 20 grains reduce the temperature to 38 deg. C. (100.5 deg. F.) this dose is sufficient. If they do not reduce it that far, a larger dose is given the next time; if they reduce it still lower, a smaller one. In this manner intermissions of the febrile process are produced as often as possible, and Liebermeister holds that the creation of complete intermissions in diseases running their course with long-continued and high grades of temperature by this remedy, is one of the principal secrets of success in their treatment.*

Any one, who has closely observed the effect of these large doses of quinine in severe cases of erysipelas, acute rheumatism, scarlatina, pleuritis, pneumonia, variola, abdominal typhus, phthisis florida, and who has measured the decrease of the temperature and seen the decided simultaneous amelioration of the circulatory and cerebral derangements following each dose, cannot long remain in doubt, that these derangements are principally caused by the effect of the high temperature on the heart and brain, and that quinine is, to use the older term, an antiphlogistic remedy in these diseases.

How far it accomplishes this result by its direct influence on the nervous system remains undecided, as its relations to the same have not yet been determined by actual experiment. That such relation exists, however, can not be denied, as its depressing effects on the nerves of sensation and voluntary motion are often too plainly perceptible, and its tonic effect on the muscular fibres of the spleen too well established. Binz's and Scharenbroich's late investigations,† however, have plainly demonstrated that quinine diminishes the capacity of the red corpuscles of the blood to bind oxygen, that it prevents the devel-

*Uber die Behandlung des Fiebers, No. 31. Sammlung Klinischer Vortraege, v., Volkman.

†Binz. Experimentelle Unter suchungen ueber das Wesen der Chininwirkung, 1868.—Med. Centralbladt, 1867, No. 52.

opment of microscopic organisms and arrests the process of fermentation and decomposition of nitrogenous substances. These qualities of the quinine, if blood-changes giving rise to fever are frequently processes more or less analogous to fermentation and decomposition, account more directly for its unquestionable efficiency in the treatment of febrile diseases.

While the veratrum viride in Europe is frequently used in the form of its alkaloid, the introduction of Norwood's excellent tincture of this plant in the practice of this country makes it unnecessary for American physicians to resort to the use of veratrine for the purpose of reducing febrile temperatures. Every American practitioner is familiar with the use of Norwood's tincture in pneumonia and other inflammatory diseases for the purpose of arresting or diminishing the intensity of the local morbid process. It is alike an invaluable remedy for reducing and controlling febrile heat in idiopathic fevers, and clear intermissions can often be established by its use without any danger to the patient. The writer has used it for the purpose of controlling the frequency of the pulse soon after its introduction by Norwood, and long before he knew anything about the medical thermometer, with the happiest effect in enteric and other low continued fevers. It should be administered often enough and in large enough doses to keep the temperature and pulse as near the normal standard as possible, without producing collapse, and five drop doses every three to four hours will prove sufficient for that purpose in a large majority of cases.

Digitalis has a very similar effect on the pulse and temperature in fevers, and is frequently used in Europe. The disagreeable cumulative symptoms however, which occasionally follow its use, and its less uniform influence on pulse and temperature, will enable us to dispense with its use when we possess a much safer and more reliable remedy in veratrum viride.

Binz's late experiments* have also placed alcohol in its different forms amongst the antipyretic remedies. He has demonstrated that it diminishes the activity of the metamorphosis in the tissues, and reduces the temperature in fever patients. When it is remembered that it furnishes at the same time hydrocarbons to the organism for combustion, and that it has a deci-

* The Therapeutical Effect of Alcohol. Atlanta Med. and Surg. Journal, vol. x, page 561.

ded antiseptic effect, its judicious use in fevers, particularly of a septic and adynamic character (pyæmia, puerperal fever, typhus, erysipelas, etc.,) is certainly frequently indicated, and frequently followed by very beneficial results.

These are the only direct antipyretic remedies now known, and therefore the only ones that can be considered in the treatment of the febrile process in its general pathological features.

The importance of nutritious diet in febrile processes of long standing is too well acknowledged by the profession generally to require more than mere mention in this paper. How far venæsection is admissible and useful in the treatment of febrile and inflammatory diseases, the writer will endeavor to consider in a future paper.

